# Adverse employment histories, work stress and self-reported depression in the French CONSTANCES study

**DOI:** 10.1093/eurpub/ckab181

**Published:** 2021-10-13

**Authors:** Hanno Hoven, Morten Wahrendorf, Marcel Goldberg, Marie Zins, Johannes Siegrist

**Affiliations:** 1 Centre for Health and Society, Medical Faculty, Institute of Medical Sociology, University of Düsseldorf, Düsseldorf, Germany; 2 INSERM, Population-based Epidemiological Cohorts Unit, UMS 011, Villejuif, France; 3 Université de Paris, France; 4 Senior Professorship on Work Stress Research, Medical Faculty, University of Düsseldorf, Düsseldorf, Germany

## Abstract

**Background:**

Job instability and disadvantaged work were shown to be associated with poor mental health, but few studies analyzed these conditions in a life course perspective. In this study, adverse employment histories are retrospectively assessed and linked to self-reported depression. Furthermore, indirect effects of later stressful psychosocial work in terms of effort-reward imbalance are investigated.

**Methods:**

With data from the French CONSTANCES cohort study of 13 716 male and 12 767 female employees aged 45 and older, we identify adverse employment histories between age 25 and 45, focussing on job discontinuity, job instability and cumulative disadvantage. Direct effects of these conditions on self-reported depression over a period of up to 5 years are analyzed, using discrete-time logistic regression. Indirect effects of stressful work at baseline are examined.

**Results:**

Moderately elevated odds ratios of self-reported depression are observed among participants with discontinued employment histories (number of unemployment periods; years out of work for men). Effort-reward imbalance at work is consistently related to elevated risk of self-reported depression and explains parts of the association between discontinuous employment and depression.

**Conclusions:**

Applying a life course perspective to occupational health research extends current knowledge. Specifically, adverse employment histories in terms of recurrent job discontinuity are related to the risk of self-reported depression. This association is partly explained by exposure to a stressful psychosocial work environment. These results can instruct labour market policies and the development of targeted worksite interventions that address disadvantage throughout entire employment trajectories.

## Introduction

For a growing proportion of employed people, working and employment conditions in modern economies are characterized by increased job instability, partly due to rapid technological change^1^ and the rise of non-standard employment.^2^ Job instability as well as working in disadvantaged occupational positions act as important determinants of stressful psychosocial experience,^3^ and these conditions are associated with elevated risks of poor mental health,^4–6^ in particular depression.^7^^,^^8^ A major shortcoming of available empirical evidence on these associations relates to the paucity of studies that analyze job instability, discontinuity and recurrent disadvantage at work over an extended period of time, assessing whole employment histories rather than using data from a single wave of data collection.^9^ Due to this shortcoming, pathways linking unstable and disadvantaged employment histories with health outcomes cannot be fully explored. In line with a leading paradigm of psychobiological stress theory, chronic exposure to psychosocial adversity exerts its negative effects on health by recurrent challenges and threats that undermine an appropriate functioning of brain and body, triggering the development of stress-related disorders.^10^ A stressful psychosocial work environment evolves from unstable chronic job exposure and is closely related to adverse chronic exposures that compromise people’s capability of successful coping with adversity.^11^ Indirect effects of work stress thus contribute to a decline in health. So far, prospective occupational health studies dealing with these indirect effects were rarely based on study designs that combine data on long-term exposure with subsequently assessed data on mediating conditions.^12^

With this contribution, we set out to overcome these limitations by analysing data that cover three time periods. The first period relates to participants’ retrospectively assessed employment histories that enable us to identify unstable and disadvantaged chronic job exposures. The process of baseline data collection defines the second time period, when participants’ current level of psychosocial stress at work is measured. Finally, the third period covers the time span from baseline data collection to final data collection on newly self-reported depression. With this approach, we examine the direct effect of chronic adversity at work on mental health as well as its indirect effect driven by subsequent exposure to a stressful psychosocial work environment. As mentioned, these indirect effects are assumed to evolve from chronic exposure and to explain, at least in part, the adverse effects on mental health.

These analyses require a distinct theoretical approach that is briefly outlined here. With the availability of retrospective information from more recently conducted occupational cohort studies collecting employment data on exposure duration, timing and its sequential character over time, as well as data on job changes and periods of unemployment, the impact on mental health attributable to recurrent changes during whole occupational careers can now be monitored. To integrate these retrospectively assessed occupational trajectories into a stress-theoretical framework, a typology of adverse employment trajectories can be developed that identifies distinct aspects of recurrent stressful experience at work. Such a typology is based on the notion of threats to fundamental material and psychosocial needs of working people, in particular the need for security and control, the need for advancement and improvement and the need of experiencing recognition, justice and fairness at work.^13–15^ At a previous occasion, we proposed the following three types of adverse employment careers: (i) trajectories characterized by high instability (e.g. temporary contracts, involuntary part-time work and forced mobility); (ii) trajectories characterized by marked discontinuity (e.g. job loss and involuntary interruptions); (iii) trajectories characterized by cumulative disadvantage (e.g. continued disadvantaged occupational position, hazard exposure and lack of promotion prospects).^16^ In all these instances, to a different extent, the needs mentioned are compromised, thus engendering chronic stressful experience. Along these lines, we defined the more proximate psychosocial stress at work in terms of the effort-reward imbalance model that focuses on the notion of violated reciprocity of exchange between efforts spent at work and rewards received in turn,^17^ thus emphasizing one of the crucial needs of working people mentioned.

Based on these theoretical notions, the following hypotheses are examined. First, we expect direct associations of reports of instability, of discontinuity, and of cumulative disadvantage with elevated risks of self-reported depression. Second, we expect that a summary measure of effort-reward imbalance at work as well as the model’s single components explain parts of the associations.

## Methods

### Data

The analyses are based on data from the French CONSTANCES project, a prospective population-based cohort focussing on occupational and environmental epidemiology.^18^ CONSTANCES started in 2012 to investigate the individual health determinants of adults who are covered by the General Health Insurance Fund (CNAM) in France (about 85% of the French population). Participants are recruited from 22 social security health screening centres (HSCs) across France. Using a random sampling stratified according to unequal inclusion probabilities and based on data from participation in previous surveys involving invitations to HSCs, participants are invited, with a participation rate of 7.3%.^19^ The sample is based on all persons aged 18–69 years covered by CNAM in the catchment areas of the CONSTANCES HSCs. Up to now, baseline data (including retrospective questionnaires) is available for 186 501 respondents. Additionally, health information is gathered and respondents are contacted annually for up to 5 years (depending on the date of their baseline assessment) to provide an update on their health status. The study was approved by bodies regulating ethical data collection in France (Comité Consultatif pour le Traitement des Informations Relatives à la Santé; Commission Nationale Informatique et Liberté), and all participants sign an informed consent.

### Study population

To analyze associations between past employments, work stress at baseline and self-reported depression at follow-up, we apply several restrictions. First, we restrict the sample to respondents who are aged 45 or older and have provided full information on their employment history between age 25 and 45 (21 years; *n* = 102 910). Furthermore, we include only respondents who have been in paid work at least once between age 25 and 45 (*n* = 96 198) and have participated in at least one follow-up period (*n* = 62 580). In order to minimize the effect of self-reported depression on adverse employment history, work stress and later reporting of depression, we excluded all respondents who have reported at baseline investigation that they have ever been diagnosed with a major depression during lifetime (*n* = 51 101; see Supplementary table S1 for the item). Finally, we restrict the sample to respondents who have been in paid work at baseline investigation and who have answered the questions on their working environment. The data restrictions result in a final sample with full available data of 13 716 men and 12 767 women.

### Measures

#### Characteristics of adverse employment history

Information on characteristics of previous employment histories is obtained from a retrospective assessment of respondents’ working careers that allows to measure the described dimensions of adverse employment history characteristics. To measure occupational instability, we take the number of temporary jobs (none, one and two or more) within the observation period (age 25–45). Discontinuous employment is measured by the number of job changes (none, one, two or more), the years out of work that have been either involuntary or voluntary (none, one to four, five or more) and the number of involuntary interruptions of the working career due to unemployment (none, one, two or more). Cumulative disadvantage is assessed by failed job promotion (promotion with sustained position thereafter, promotion without sustained position and no job promotion) and the main occupational position during the observation period. Occupational position is measured by the French classification scheme of occupations (‘Professions et catégories socioprofessionelles’ converted to seven different classes using the European Socio-Economic Classification scheme).^20^ An additional category captures workers who have not specified their main occupational position. To measure the main occupational position, we assess the occupational position from the respondents’ longest job. If a respondent had not only one main job but two or more main jobs with exactly the same length, we prioritize the most recent one.

#### Baseline working conditions

Respondents’ working conditions at baseline are assessed by psychosocial working conditions that are measured by the short version of the effort-reward imbalance questionnaire, previously validated in this cohort.^21^ We include high efforts, low rewards and the ratio of high efforts and low rewards (ER-ratio) adjusted for the unequal number of items as extrinsic components and over-commitment as intrinsic component into our analysis. Based on the final sample, we derive tertiles for each measure, where high work stress and high over-commitment is assumed in the highest tertile of each distribution.

#### Self-reported depression

At each annual follow-up period, respondents are asked if they have had a depression during the past 12 months, regardless of whether or not there has been a work interruption or a treatment/diagnosis by a doctor (see Supplementary table S1 for the item). We combine the information on self-reported depression for each follow-up period. This results in up to five binary indicators of self-reported depression for each respondent (depending on the number of the individual follow-up periods).

#### Additional variables

We additionally include sex, age (linear and squared), assessment centre (centres with <250 cases have been regrouped into one joint category), partnership (living with spouse and living without spouse) and education as potential confounders and income as potential mediator into the analyses. Income is measured by the household equivalent income divided into tertiles (based on the final sample). Due to the high number of missing values, we include a fourth category entitled ‘answer refused’ (*n* = 1096). Education is assessed by the International Standard Classification of Educational Degree (ISCED) regrouped into low (pre-primary, primary or lower secondary education), medium (upper secondary or post-secondary education) and high (first and second stage of tertiary education) education. All additional variables are measured at baseline investigation.

#### Statistical analysis

**Figure 1:**
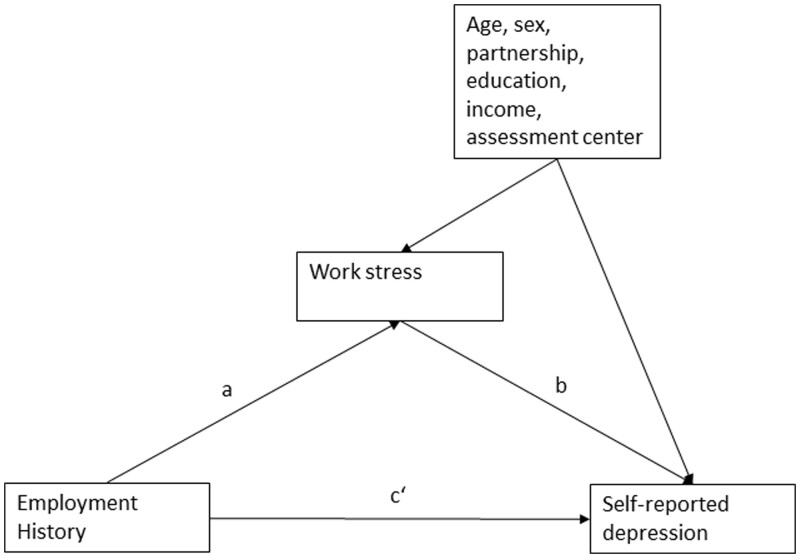
Pathways between Employment History, Work stress and self-reported depression

In a first step, the sample distribution is described for all baseline characteristics, including information on current working conditions and the retrospectively assessed measures of previous employment history (age 25–45) (see table 1). The event history of self-reported depression is presented in a Lifetable (see Supplementary table S2), reporting key characteristics of the follow-up data. To estimate the risk of self-reported depression, we rearrange the data from a person-level dataset to a person-period dataset, and we assign for each respondent as many observations as he or she participated in follow-up periods (resulting in a maximum of five person-period observations for each respondent). In order to additionally adjust for potential confounders, we apply discrete-time logistic regressions for the analysis of event histories.^22^ With these models, we include a categorical variable of the time intervals (the time from one follow-up period to another) into the regression models, enabling the estimation of odds for self-reported depression separately for each time interval (see table 2). In a final step, we decompose the effects of past discontinuous employment history on self-reported depression into direct and indirect (via high effort-reward imbalance) effects (see table 3). We only report results for the number of unemployment periods, because the regression models of the previous step have shown that only unemployment is linked to later self-reported depression for both men and women (see table 2). First, we estimate the direct effect from employment history to mental health (path c). Second, we estimate paths from employment history to subsequent ERI separately for each category of employment history (paths a_1_ and a_2_). Third, we estimate a path from ERI to mental health (path b). The indirect effect for each category of employment history is obtained by the product of a_1_ and b, and a_2_ and b, respectively. The paths of the pathway model are summarized in figure 1. Pathway models are estimated using logistic regressions in the generalized structural equation framework (gsem) of Stata. Confidence intervals for the indirect effects are derived from bootstrapping procedures with 3000 repetitions. To facilitate interpretation, we additionally report the proportion of the indirect effect by dividing the indirect effect (numerator) by the sum of the indirect and direct effect (denominator) using the formula: $\frac{\left(a_{1}\times b_{1})+(a_{2}\times b_{2}\right)}{\left(\left(a_{1}\times b_{1})+(a_{2}\times b_{2}\right)\right)+c\prime }$. This formula has been suggested to provide reasonable results in a recent simulation study.^23^ We use Stata 16.1 for data preparation and statistical analysis.

**Table 1:** Sample description: observations (No.) and percentages (Col. %), *n* = 26 483

		Men		Women	
	Categories or range	No.	Col % or mean (SD)	No.	Col % or mean (SD)
Age	45–72	13 716	53.1 (5.3)	12 767	52.9 (5.1)
Partnership	Living with spouse	11 336	82.7	9301	72.9
	Not living with spouse	2380	17.4	3466	27.2
Education	Low	1086	7.9	1086	8.0
	Medium	5215	38.0	4403	34.5
	High	7415	54.1	7344	57.5
Number of temporary jobs	None	11 703	85.3	10 204	80.0
	One	1771	12.9	2150	16.8
	2+	242	1.8	413	3.2
Number of job changes	None	3514	25.6	3926	30.8
	One or two	6012	43.8	5479	42.9
	three or more	4190	30.6	3362	26.3
Years out of work	None	8797	64.1	6227	48.8
	1–5	3686	26.9	3685	28.9
	6+	1233	9.0	2855	22.4
Number of unemployment periods	None	12 280	89.5	10 822	84.8
	One	1181	8.6	1580	12.4
	2+	255	1.9	365	2.9
Job promotion	No promotion	10 206	74.4	10 221	80.1
	Promotion with sustained position	2968	21.6	2212	17.3
	Promotion without sustaining position	542	4.0	334	2.6
Main occupational position	Large employers, higher managers and professionals	2525	18.4	1295	10.1
	Lower managers and professionals	2921	21.3	4501	35.3
	Intermediate employee	2034	14.8	2033	23.7
	Small employers and self-employed	241	1.8	122	1.0
	Lower grade white collar workers	358	2.6	1045	8.2
	Skilled workers	1476	10.8	201	1.6
	Semi- or unskilled workers	927	6.8	403	3.2
	Main position unknown	3234	23.6	2178	17.1
Income	Low	4418	32.2	4339	34.0
	Medium	4799	35.0	4305	33.7
	High	3987	29.1	3539	27.7
	Answer refused	512	3.7	584	4.6
High effort	No	10 420	76.0	9244	72.4
	Yes	3296	24.0	3523	27.6
Low reward	No	9423	68.7	8506	66.6
	Yes	4293	31.0	4261	33.4
High ER-ratio	No	10 061	73.4	8702	68.2
	Yes	3655	26.7	4065	31.8
High Over-commitment	No	9741	71.0	8313	65.1
	Yes	3975	29.0	4454	34.9
Self-reported depression	No	13 314	97.1	12 248	95.9
	Yes	402	2.9	519	4.1

**Table 2:** Adverse employment history and self-reported depression. Discrete-time logistic regressions, odds ratios (95% confidence intervals) for men (33 818 person-period observations) and women (31 723 person-period observations)

		Men				Women			
		M1		M2		M1		M2	
	None (ref)	–	–	–	–	–	–	–	–
Number of temporary jobs	One	1.40	(1.08/1.83)	1.35	(1.03–1.76)	1.24	(0.99/1.54)	1.19	(0.95/1.48)
	2+	1.67	(0.91/3.07)	1.50	(0.81–2.77)	1.05	(0.64/1.71)	0.98	(0.60/1.61)
Number of job changes	None			–	–			–	–
	One or two	0.89	(0.70/1.14)	0.90	(0.70/1.14)	0.96	(0.77/1.18)	0.96	(0.78/1.19)
	three or more	0.94	(0.72/1.22	0.94	(0.72/1.22)	1.28	(1.02/1.60)	1.28	(1.02/1.61)
Years not in paid work	None (ref)			–	–			–	–
	1–5	1.33	(1.07/1.66)	1.33	(1.06/1.66)	1.24	(1.01/1.52)	1.22	(0.99/1.49)
	6+	1.71	(1.26/2.31)	1.46	(1.07/2.00)	1.16	(0.93/1.45)	1.09	(0.87/1.37)
Number of unemployment periods	None (ref)			–	–			–	–
	One	1.50	(1.11/2.03)	1.37	(1.01/1.86)	1.42	(1.12/1.79)	1.33	(1.05/1.69)
	2+	1.56	(0.85/2.87)	1.34	(0.73/2.48)	1.38	(0.87/2.20)	1.28	(0.80/2.04)
Job promotion	No promotion (ref)			–	–			–	–
	Promotion with sustained position	0.93	(0.73/1.19)	0.95	(0.74/1.21)	1.16	(0.93/1.45)	1.14	(0.91/1.42)
	Promotion without sustaining position	1.37	(0.87/2.14)	1.36	(0.87/2.13)	1.74	(1.12/2.69)	1.68	(1.09/2.61)
Main occupational position	Large employers, higher managers and professionals (ref)	–	–	–	–	–	–	–	–
	Lower managers and professionals	1.22	(0.89/1.67)	1.14	(0.83/1.57)	0.96	(0.69/1.33)	0.88	(0.63/1.21)
	Intermediate employee	1.10	(0.77/1.56)	0.96	(0.66/1.40)	1.23	(0.89/1.72)	1.02	(0.70/1.47)
	Small employers and self-employed	0.75	(0.30/1.86)	0.59	(0.23/1.50)	0.86	(0.31/2.40)	0.77	(0.27/2.18)
	Lower grade white collar workers	1.39	(0.76/2.53)	0.94	(0.50/1.78)	1.05	(0.69/1.60)	0.79	(0.49/1.26)
	Skilled workers	1.05	(0.71/1.55)	0.79	(0.50/1.23)	1.23	(0.60/2.53)	1.00	(0.48/2.10)
	Semi- or unskilled workers	1.28	(0.83/1.97)	0.89	(0.55/1.45)	1.27	(0.74/2.18)	0.94	(0.52/1.68)
	Main position unknown	1.01	(0.74/1.40)	0.89	(0.64/1.25)	1.27	(0.90/1.79)	1.10	(0.77/1.58)
High effort	No (ref)	–	–	–	–	–	–	–	–
	Yes	1.57	(1.28/1.94)	1.67	(1.35/2.07)	1.73	(1.45/2.07)	1.81	(1.51/2.17)
Low reward	No (ref)	–	–	–	–	–	–	–	–
	Yes	2.11	(1.73/2.57)	1.99	(1.63/2.44)	2.26	(1.89/2.68)	2.15	(1.80/2.57)
High ER-ratio	No (ref)	–	–	–	–	–	–	–	–
	Yes	1.96	(1.60/2.39)	1.92	(1.57/2.35)	2.20	(1.85/2.62)	2.17	(1.82/2.59)
High Over-commitment	No (ref)	–	–	–	–	–	–	–	–
	Yes	1.82	(1.49/2.22)	1.94	(1.58/2.37)	1.97	(1.66/2.35)	2.15	(1.80/2.57)

Based on separate logistic regression models. M2 is adjusted for assessment centre, age, age squared, education, partnership and income.

**Table 3:** Unemployment and self-reported depression for men and women. Direct and indirect (via effort-reward imbalance) effects (65 541 person-period observations). Unstandardized regression coefficients (95% CI)

		Direct effects		Indirect effects	
Number of unemployment periods	None (ref)				
	One	0.28	(0.09/0.46)	0.11	(0.07/0.16)
	2+	0.28	(−0.10/0.65)	0.02	(−0.06/0.11)
	Proportion of indirect effect	19.6%			

Based on logistic regression models, adjusted for sex, age, age squared, assessment centre, partnership, education and income. Confidence intervals for the indirect effects are based on bootstrapping procedures with 3000 replications.

## Results

In table 1, we give a sample description separately for men and women. We see that men have worked in more advantaged occupational positions and have had more continuous working careers than women, with less unemployment interruptions but more job changes. Women in contrast have a higher probability of a history with temporary jobs. Men report slightly better psychosocial working conditions, and the proportion of workers with an imbalance between efforts and rewards is higher among women than among men. About 4% of women and 2.9% of men report a depression during follow-up.

Details of the distribution of self-reported depression are presented in Supplementary table S2. In summary, for the 13 716 men, data on 33 818 person-periods are available with more than half of the men providing data for at least two follow-up periods (mean follow-up: 2.1 periods). The 12 767 women provided data on 31 723 person-periods (mean follow-up: 2.1 periods). More women than men have reported a depression (519 vs. 402) and the probability of ‘survival’ without a self-reported depression during follow-up is higher for men than for woman.

Results of the first hypothesis on associations between adverse employment histories and self-reported depression are reported in table 2, supporting the hypothesis to a limited extent only. Elevated odds ratios of self-reported depression are observed for specific categories of the three indicators of a discontinued employment career [years out of paid work (for men only), number of unemployment periods (for men and women who experienced one period) and number of job changes (women with three or more changes)]. Instability, as measured by number of temporary jobs, is inconsistently related to self-reported depression as effects are significant only for men who experienced this condition once. Our two measures of cumulative disadvantage do not seem to predict self-reported depression (with the exception of promotion without sustaining the position for women). When considering the main categories of occupational position, we observe no effects. Distinct from these selective relationships, effort-reward imbalance at work is consistently associated with elevated risks of self-reported depression, i.e. among men and women, across all model components, and for the estimate of ER-ratio even if adjusting for the main effects of effort and reward (not shown). Importantly, estimates of employment history and effort-reward imbalance do not alter if considering and the impact of potential confounders in multivariable regressions, including income.

Turning to the second hypothesis, the findings on direct and indirect effects on self-reported depression are displayed in table 3. For those who experienced one period of unemployment, parts of the association between previous employment history and self-reported depression are explained by indirect effects of elevated levels of subsequent work stress (as measured by effort-reward imbalance). The effects of number of unemployment periods are thus partly explained by indirect effects of effort–reward imbalance.

## Discussion

In this study, we investigated associations between retrospectively assessed characteristics of adverse employment histories, working conditions measured at baseline (effort-reward imbalance) and new self-reported depression, using data from a large French cohort study. Results suggest that participants who previously experienced unemployment periods, temporary employment or years out of paid work (for men) are at elevated risk of self-reported depression. Additional aspects of adverse employment trajectories (disadvantaged occupational position, frequent job changes and failed job promotion) were however not related to self-reported depression. As a further finding, we observe inconsistent indirect effects of work stress. Only a limited amount of the link between employment characteristics is explained by subsequent psychosocial stress at work.

Our findings are in line with previous support of the notion that recurrent job instability (temporary employment) and job discontinuity (periods of unemployment and years out of work) affect health in the long run by evoking chronic stress responses and that these associations are more consistent among men than among women.^24^ These exposures elicit feelings of unpredictability and uncontrollability, two important determinants of the intensity of stressful experience.^25^ Unexpectedly, we did not see an established social gradient of poor mental health in this population, leaving participants with lower occupational positions at higher risk.^26^ One reason may be that the disadvantaged occupational positions were underrepresented in the final study sample (see table 1). Another explanation relates to the methodology of assessing mental health where self-report data are vulnerable to a systematic reporting bias according to socio-economic position (see below). Clearly, additional research is needed to tackle the observed discrepancy.

In addition to confirming previous associations of effort–reward imbalance at work with poor mental health,^8^ our study adds to research on the role of psychosocial working conditions explaining parts of the social gradient in health.^27^ Studies that examined retrospectively assessed job careers with future health and that included comparable pathways are scarce. The findings of this study illustrate the importance of a life course perspective. As such, long-standing disadvantaged occupational trajectories seem to promote further adversities in the sense of a pathway model with early disadvantages impacting later experiences that consequently lead to poor health.^28^ Frustration at work, triggered by an unrewarding environment (low gain received despite high efforts expended) further aggravates experienced adversity during the working career, in line with work stress theory.^17^

On a final note, our study results instruct the development of intervention measures towards improving the mental health of socially disadvantaged workers. They suggest, first, that targeted structural reductions of career adversities, starting at early stages of employment trajectories, can exert beneficial effects on mental health later on. Second, they point to conditions within these trajectories that aggravate the health risk and thus call for additional intervention efforts. Further research may elaborate these findings by more comprehensively investigating exposure–mediator interactions.^29^

Our study suffers from several limitations. First, no clinically validated measures of depression were available for this analysis. Self-reported data suffer from restricted validity and reporting bias. Symptoms of depression are often not clearly distinguished, and self-reported data carry a considerable risk of misclassification.^30^^,^^31^ Reporting bias may also in part explain the lack of an observed social gradient of self-reported depression, given the fact that participants with lower socio-economic position were shown to underreport mental symptoms.^32^^,^^33^ This lack of a social gradient is in obvious contrast to established evidence.^16^^,^^26^ A second limitation concerns the retrospective assessment of employment careers at baseline. In fact, retrospective data may generally be vulnerable to recall bias. The assessment of employment history and work stress at the same time may additionally yield common method biases, for instance due to consistency motifs of the respondents.^34^ Yet, retrospective employment history data have been shown to provide reliable and valid information when comparing to prospective birth cohort and administrative data.^35–37^ Third, work stress has been measured only once at baseline. To prevent reverse causation, it would have been desirable however, to include information on previous work stress (e.g. before age 25) and on the change of work stress during follow-up. Finally, the operational definition of our proposed typology of adverse employment careers calls for further in-depth elaboration, for instance by applying latent growth modelling.^38^ For instance, an appropriate measurement of frequent job change requires an additional differentiation of undesirable aspects of this change. For temporary jobs, the career stage may be essential, as temporary jobs at early career stage are more common, whereas, at older age, they are experienced as threatening events.

These limitations are balanced by several strengths. First, this is one of the rare investigations in occupational epidemiology that combine prospective data (on health) with retrospectively assessed employment conditions (as determinants), and that include indirect effects (assessed at baseline). Even more important, the predicting factors are rooted in a conceptual framework of life course social inequalities in health with elaborated links between occupational conditions, adverse psychosocial work environments and disadvantaged health.^39^ As a second strength, this study provides data collected with high quality standards on a large sample of employed men and women in midlife and early old age in France. The timespan assessing employment careers covers two decades, and the annual follow-up of reports of depression over 5 years reduces the risk of reverse causation. In summary, the richness of information provided by this study design and by the breath of socio-economic, occupational and health-related indicators included in the analysis contributes to the innovative aspects of this investigation.

In conclusion, this is one of the first studies demonstrating associations of retrospectively assessed adverse employment careers, in terms of recurrent job instability and job discontinuity, with self-reported depression in a large sample of employed men and women in France. To some extent, these associations are driven by indirect effects of subsequent stressful psychosocial work. If further confirmed, our results can instruct labour market policies as well as the development of targeted worksite intervention approaches that identify stages of increased susceptibility to poor mental health among workers exposed to adverse employment careers.

## Supplementary data

Supplementary data are available at *EURPUB* online.

## Funding 

This work was supported by funding from the German research foundation (Deutsche Forschungsgemeinschaft, project number: SI 236/16-1 and WA 3065/5-1). The CONSTANCES Cohort Study was supported and funded by the Caisse nationale d’assurance maladie des travailleurs salariés (CNAM). The Constances Cohort Study is an ‘Infrastructure nationale en Biologie et Santé’ and benefits from a grant from ANR (ANR-11-INBS-0002). Constances is also partly funded by MSD, AstraZeneca, L’Oréal and Lundbeck.

*Conflicts of interest*: None declared.

**Key points**

- Few studies analyzed associations between adverse employment history and poor mental health applying a life course perspective.
- In this study, we link retrospectively assessed employment history with self-reported depression and indirect effects of stressful psychosocial work.
- Adverse employment histories in terms of recurrent job discontinuity are related to elevated risk of self-reported depression.
- This association is partly explained by indirect effects of subsequent stressful psychosocial work as measured by effort-reward imbalance.

## Supplementary Material

ckab181_Supplementary_Data
