# Supplementary material for: Adverse employment histories, work stress and self-reported depression in the French CONSTANCES study
Source: Eur J Public Health. 2021 Oct 13;31(6):1230–6. doi: 10.1093/eurpub/ckab181 (PMC8995100; doi:10.1093/eurpub/ckab181)
Supplement: ckab181_Supplementary_Data [file ckab181_supplementary_data.docx]

Supplementary Table S1: Items of self-reported depression

| Baseline | Liste des Antécédents Médicaux Personnels  Dépression traitée: oui/non  [List of Personal Medical History  Treated Depression: yes/no] |
| --- | --- |
| Follow-up | Voici une liste de problèmes de santé. Indiquez ici ceux dont vous souffrez ou avez souffert au cours des 12 derniers mois (qu’il y ait eu ou non un arrêt de travail, qu’il y ait ou non un traitement).  -Dépression  [Here is a list of health problems. Indicate here which ones you have or have had in the last 12 months (whether or not you have been off work, whether or not you have been treated).  -Depression] |

Note: In the original questionnaire the respondents had the option to choose among a variety of diseases (e.g. hypertension, asthma). We focused on self-reported depression.

Supplementary Table S2: Lifetable for self-reported depression by sex, n=26,483

| Interval | Number of respondents entering interval | Respondents with self-reported depression | Respondents censored in interval | Discrete-time Hazard | Probability of Survival |
| --- | --- | --- | --- | --- | --- |
| **Men** | | | | | |
| [0,1] | 13,716 | 247 | 3,822 | 0.0180 | 0.9820 |
| [1,2] | 9,647 | 112 | 3,342 | 0.0116 | 0.9706 |
| [2,3] | 6,193 | 32 | 2,840 | 0.0052 | 0.9656 |
| [3,4] | 3,321 | 9 | 2,371 | 0.0027 | 0.9630 |
| [4,5] | 941 | 2 | 939 | 0.0021 | 0.9609 |
| Total | 33,818 | 402 | 13,314 |  |  |
| **Women** | | | | | |
| [0,1] | 12,767 | 313 | 3,431 | 0.0245 | 0.9755 |
| [1,2] | 9,023 | 132 | 3,122 | 0.0146 | 0.9612 |
| [2,3] | 5,769 | 51 | 2,484 | 0.0088 | 0.9527 |
| [3,4] | 3,234 | 22 | 2,282 | 0.0068 | 0.9462 |
| [4,5] | 930 | 1 | 929 | 0.0011 | 0.9452 |
| Total | 31,723 | 519 | 12,248 |  |  |
